# GITRL on dendritic cells aggravates house dust mite-induced airway inflammation and airway hyperresponsiveness by modulating CD4^+^ T cell differentiation

**DOI:** 10.1186/s12931-020-01583-x

**Published:** 2021-02-08

**Authors:** Yaping Wang, Kou Liao, Bo Liu, Chao Niu, Wenjing Zou, Lili Yang, Ting Wang, Daiyin Tian, Zhengxiu Luo, Jihong Dai, Qubei Li, Enmei Liu, Caihui Gong, Zhou Fu, Ying Li, Fengxia Ding

**Affiliations:** 1 Department of Respiratory Medicine, Children’s Hospital of Chongqing Medical University, Yuzhong District, No. 136, Zhongshan 2nd Road, Chongqing, 400014 China; 2 National Clinical Research Center for Child Health and Disorders, Ministry of Education Key Laboratory of Child Development and Disorders, China International Science and Technology Cooperation Base of Child Development and Critical Disorders, Chongqing Key Laboratory of Pediatrics, Children’s Hospital of Chongqing Medical University, Chongqing, People’s Republic of China; 3 Department of Cardiothoracic Surgery, Children’s Hospital of Chongqing Medical University, Chongqing, People’s Republic of China

**Keywords:** GITRL, Dendritic cells, Asthma, Th1/Th2, Th17/Treg

## Abstract

**Background:**

Glucocorticoid-induced tumor necrosis factor receptor family-related protein ligand (GITRL) plays an important role in tumors, autoimmunity and inflammation. However, GITRL is not known to modulate the pathogenesis of allergic asthma. In this study, we investigated whether regulating GITRL expressed on dendritic cells (DCs) can prevent asthma and to elucidate its mechanism of action.

**Methods:**

In vivo, the role of GITRL in modulating house dust mite (HDM)-induced asthma was assessed in adeno-associated virus (AAV)-shGITRL mice. In vitro, the role of GITRL expression by DCs was evaluated in LV-shGITRL bone marrow dendritic cells (BMDCs) under HDM stimulation. And the direct effect of GITRL was observed by stimulating splenocytes with GITRL protein. The effect of regulating GITRL on CD4^+^ T cell differentiation was detected. Further, GITRL mRNA in the peripheral blood of asthmatic children was tested.

**Results:**

GITRL was significantly increased in HDM-challenged mice. In GITRL knockdown mice, allergen-induced airway inflammation, serum total IgE levels and airway hyperresponsiveness (AHR) were reduced. In vitro, GITRL expression on BMDCs was increased after HDM stimulation. Further, knocking down GITRL on DCs partially restored the balance of Th1/Th2 and Th17/Treg cells. Moreover, GITRL stimulation in vitro inhibited Treg cell differentiation and promoted Th2 and Th17 cell differentiation. Similarly, GITRL mRNA expression was increased in the peripheral blood from asthmatic children.

**Conclusions:**

This study identified a novel role for GITRL expressed by DCs as a positive regulator of CD4^+^ T cells responses in asthma, which implicates that GITRL inhibitors may be a potential immunotherapy for asthma.

## Introduction

Asthma is one of the most prevalent chronic respiratory diseases and affects more than 300 million people worldwide [1]. It is characterized by airway inflammation and airway hyperresponsiveness (AHR). It is caused by immune dysfunction that is predominantly affected by increased effector T cell subsets and decreased regulatory T cells (Tregs) [2]. T helper 2 (Th2) cells are thought to mediate eosinophilic asthma by secreting cytokines, such as IL-4, IL-5, IL-13 [3]. In contrast, Th1 cells mainly act as negative regulators of allergic inflammation by inhibiting Th2 responses [4]. Th17 cells are thought to be associated with severe, steroid-resistant asthma [5], which is often characterized by neutrophilic infiltration. However, Tregs downregulate the immune responses and are considered to be important for maintaining immune homeostasis [6]. Therefore, decreasing effector T cells while increasing Tregs may restore the immune balance of asthmatics.

Dendritic cells (DCs) are professional antigen-presenting cells (APCs) that play an important role in the development of immune responses to environmental triggers [7]. Costimulatory molecules and cytokines of DCs and its surrounding cells affect the outcome of Th cell differentiation in asthma [8]. Glucocorticoid-induced tumor necrosis factor receptor-related receptor (GITR) is a member of the TNF receptor superfamily [9]. Its ligand, GITRL, is mainly expressed on various APCs, such as DCs, B cells, macrophages and endothelial cells [10, 11]. GITR/GITRL plays a critical role in diverse immune processes including inflammation, transplantation, allergy, and autoimmunity [12]. Studies have suggested that the GITR/GITRL interaction can inhibit the suppressive function of Tregs and promote the proliferation of effector T cells [13–18]. A study also showed that vaccination with bone marrow dendritic cells (BMDCs) overexpressing GITRL can significantly inhibit tumor growth accompanied by a significant decrease in Tregs [19]. Furthermore, another study suggested that treatment with soluble GITRL can reduce the inhibitory effect of tumor-infiltrating Tregs and restore the proliferation of CD4^+^CD25^−^ T cells [20]. Thus, GITR/GITRL can regulate inflammation and immunity by inhibiting the suppressive function of Tregs. On the other hand, a study showed increased GITR/GITRL expression in lung tissues of ovalbumin-induced asthmatic mice [21]. In addition, GITR activation aggravates AHR and serum IgE responses in asthmatic mice and increases the production of Th2 cytokines [22, 23]. These findings imply that GITR/GITRL signaling may play a role in asthma by regulating immunity. However, limited data exist regarding the mechanism of GITRL in allergen-mediated asthma and the therapeutic effect of blocking GITRL on DCs in asthma.

In our study, we mainly use adeno-associated virus (AAV)-shGITRL and LV-shGITRL to knockdown the expression of GITRL on the surface of DCs in vivo and in vitro, and then detect the differentiation of CD4^+^ T cells and its effect on the asthma phenotype, which provides a basis for immunotherapy of asthma and has important clinical significance.

## Materials and methods

### Mice

Female C57BL/6 mice were purchased from the Experimental Animal Center of Chongqing Medical University (Chongqing, China) and maintained under specific pathogen-free conditions. For experiments, age-matched mice 4 to 6 weeks of age were used. This study was approved by the Institutional Animal Care and Committee (IACUC), which is accredited by the Association for Assessment and Accreditation of Laboratory Animal Care International, China and Experimental Animal Committee of the Chongqing Medical University (license numbers: SYXK (Yu) 2017–0012). All animal experiments were performed in accordance with the guidelines of Chinese Council on Animal Care and Use.

### GITRL knockdown by a recombinant AAV delivery approach

AAV6-green fluorescent protein (GFP) and AAV6-shGITRL were constructed (GeneChem, Shanghai, China). The administration procedures were performed according to previous studies [24, 25]. In brief, 5 × 10^10^ physical particles of AAV6 in 60 μl of normal saline were administered to 4-week-old C57BL/6 mice by the intranasal route. Two weeks later, the transfected mice were used to establish the allergic asthma model.

### HDM sensitization and challenge model

For house dust mite (HDM)-induced asthma, mice were sensitized by 20 μg of HDM intranasally (Greer, Los Angeles, CA, USA) on day 0 and day 14 and then challenged by intranasal HDM (20 μg) on days 21, 23, 25, 27 and 29. Mice in the control group were sensitized and challenged with normal saline instead of HDM. Endpoints were analyzed on day 31.

### Lung histopathology and immunohistochemistry

Left lung tissues were fixed in 4% buffered formalin and embedded in paraffin. Sagittal sections were cut at a thickness of 4 μm for hematoxylin–eosin staining and immunohistochemical (IHC) analyses. The severity of inflammation in lung tissues was evaluated on a 0–3 scale defined as follows: 0 points for no inflammatory reaction; 1 point for mild inflammatory reaction, punctate inflammatory cells infiltration in bronchial or vascular walls and alveolar septum; 2 points for moderate inflammatory reaction, patchy inflammatory cells infiltration in bronchial or vascular walls and alveolar septum with a wetted area that is less than 1/3 of the cross-sectional area of lung; and 3 points for a severe inflammatory reaction, diffuse inflammatory cell infiltration in the bronchial or vascular walls and alveolar septum with an infiltration area of 1/3–2/3 of the cross-sectional area of the lung. For IHC staining, rabbit anti-GITRL (1:200; GeneTex, Irvine, CA, USA) primary antibody was used. GITRL-positive cells were stained brown with DAB. The integral optical density (IOD) of the positive areas was analyzed using Image-Pro Plus 6.0 software.

### Bronchoalveolar lavage and cell counting

The left bronchus of the mice was ligated, and the right lung was rinsed three times with sterile phosphate buffered saline (PBS; 0.5 ml) to collect bronchoalveolar lavage fluid (BALF). After centrifugation of the BALF, the obtained cells were lysed with red blood cell lysis buffer and then counted with a cell counter. For the eosinophil count, the remaining cells were stained with Wright-Giemsa stain (Jiancheng Techno Co, Nanjing, China) according to the protocol. The proportion of eosinophils was obtained by counting at least 200 cells, and the total number of eosinophils was calculated.

### Detection of total IgE

Blood samples were obtained and centrifuged at 2500 rpm for 15 min at room temperature. The supernatants were used for IgE determinations. Total serum IgE levels were detected with a murine IgE ELISA kit (NeoBioscience, Shenzhen, China) according to the protocol.

### Measurement of AHR

Mice were anesthetized with 2% pentobarbital sodium, and lung resistance to different doses of methacholine was measured using an invasive pulmonary function instrument (EMKA Technologies, Paris, France) within 24 h after the last HDM challenge. Mice were mechanically ventilated following tracheal cannulation in whole-body plethysmography chambers with a constant inspiratory flow before nebulization with increasing doses of methacholine (0–50 mg/ml). Lung resistance at each concentration was calculated.

### Western blotting

Total proteins from lung tissues or BMDCs were extracted using the total protein extraction kit (KeyGen BioTECH, Jiangsu, China). Protein concentrations were routinely determined by the bicinchoninic acid protein assay reagent. Equal amounts of protein were subjected to 10% sodium dodecyl sulfate polyacrylamide gel electrophoresis and then transferred to polyvinylidene fluoride membranes. Membranes were blocked with 5% skimmed milk powder solution and then incubated with 1:1000 rabbit anti-GITRL antibody (GeneTex, Irvine, CA, USA) as a primary antibody and 1:5000 diluted goat anti-rabbit IgG as a secondary antibody (ProteinTech, Wuhan, China). Band intensities were quantified using Image Lab 6.0 software, and values were normalized to β-actin.

### Generation of BMDCs

Bone marrow (BM) cells were obtained by flushing the femurs and tibias of C57BL/6 mice with RPMI 1640 medium as previously described [26, 27]. Briefly, BM cells were cultured in RPMI 1640 medium supplemented with GM-CSF (20 ng/ml) and IL-4 (10 ng/ml), 10% FBS, glutamine, and penicillin–streptomycin for 6 days. At day 7, nonadherent cells were collected as BMDCs, and more than 80% of the cells expressed characteristic DC-specific markers as determined by flow cytometry.

### Transduction of BMDCs with lentiviral vectors (LV)

BMDCs were transfected with control or GITRL-specific shRNAs (GeneChem, Shanghai, China). The sequences targeted by shRNAs were as follows: shGITRL-1, 5′-ccCTTCGTAGTACAGATATAT-3′; shGITRL-2, 5′-gcCAAGTGATTCCTGTGGATA-3′; and shGITRL-3, 5′- gtTTGAACTATCATCCTCAAA-3′. Shcontrol, which did not target any known gene, was used as control. BM cells were seeding in RPMI-1640 medium supplemented with GM-CSF and IL-4 for 2 days and then transduced with shcontrol or shGITRL at a multiplicity of infection of 100. After 24 h of transduction, transduced BM cells were washed and seeded for DC differentiation in the presence of GM-CSF and IL-4 and used at least 4–6 days later. GFP expression in the transduced BMDCs was observed by fluorescence microscopy 4–6 days after transduction, and GFP-positive cells were detected by flow cytometry.

### Coculture of BMDCs and splenocytes

BMDCs were collected on days 7–8 and adjusted to a concentration of 5 × 10^4^ cells/ml. Splenocytes were obtained from the spleens of C57BL/6 mice by mechanical disruption, 300 mesh stainless steel screen filtration and Ficoll density gradient purification. Splenocytes and BMDCs were mixed at a ratio of 10:1 and cocultured in 24-well plates for 72 h with or without HDM (25 μg/ml) stimulation. Th1, Th2, Th17 and Treg cells were determined by flow cytometry based on surface markers and intracellular cytokine expression. The culture supernatant was harvested for cytokine detection.

### Immunofluorescence detection

Frozen 10-µm lung tissue sections were used for immunofluorescence. Lung tissue sections and BMDCs were fixed with 4% paraformaldehyde, permeabilized with Triton X100, and blocked with 5% bovine serum albumin. Lung tissue sections and BMDCs were then stained with rabbit anti-GITRL (1:200; GeneTex, Irvine, CA, USA) as a primary antibody and stained with cy3-conjugated goat anti-rabbit (1:200; Proteintech, Wuhan, China) as a secondary antibody. Nuclei were counterstained with 40, 6-diamidino-2-phenylindole (Beyotime, Shanghai, China). Images were captured under a fluorescence microscope (Nikon, Tokyo, Japan) and analyzed with ImageJ software.

### Stimulation of splenocytes with GITRL in vitro

Splenocytes from C57BL/6 mice were stimulated with anti-CD3 (1 μg/ml, BioLegend, USA) or anti-CD3 (1 μg/ml, BioLegend, USA) plus GITRL (1 μg/ml, MedChemExpress, China) for 48 h before harvesting. Th1, Th2, Th17 and Treg cells were determined by flow cytometry based on surface markers and intracellular cytokine expression. The culture supernatant was harvested for cytokine detection.

### Flow cytometry

Mouse lung tissue was digested with collagenase to generate a single-cell suspension. Lung cells or cultured cells were stained with CD11c (anti-CD11c-PE-Cy5.5, e Bioscience, USA), MHCII (anti-MHCII-PE, BD Pharmingen, USA) and GITRL (anti-GITRL-BV421, BD Pharmingen, USA) to identify CD11c^+^MHCII^+^GITRL^+^ DCs. For the analysis of Tregs, lung cells or cultured cells were first stained for CD4 (anti-CD4-APC-Cy7, BD Pharmingen, USA) and CD25 (anti-CD25-PE, BD Pharmingen, USA) and then fixed and permeabilized for intracellular Foxp3 (anti-Foxp3-APC, e Bioscience, USA) staining. For detecting Th1, Th2 and Th17 cells, lung cells or cultured cells were stimulated with phorbol 12-myristate 13-acetate (50 ng/ml) and ionomycin (1 μg/ml) for 6 h in the presence of Golgi Stop solution. After stimulation, cells were incubated with surface markers for CD4 (anti-CD4-FITC, BD Pharmingen, USA), permeabilized and stained for the intracellular cytokines IL-4 (anti-IL-4-APC, BD Pharmingen, USA), IFN-γ (anti-IFN-γ-BV421, BD Pharmingen, USA) and IL-17 (anti-IL-17-PE, BD Pharmingen, USA). Data were acquired using FACS Canto II (BD Biosciences, USA) and analyzed with FlowJo software.

### Asthma patients and sample collection

Twelve children with asthma admitted to the Children’s Hospital of Chongqing Medical University (Chongqing, China) were enrolled in this study. Patients were selected based on the inclusion and exclusion criteria for the diagnosis of childhood asthma. The asthma patients we included were first diagnosed with asthma and did not receive glucocorticoid treatment. Children without respiratory diseases were enrolled as a control group. Peripheral blood samples were collected and used to prepare total RNA. The study was approved by the Human Research Ethics Committee of Chongqing Medical University (permit number: 2018-148), and written informed consent was obtained from guardians of all subjects before participation.

### Quantitative RT-PCR

Total RNA from peripheral blood samples and lung tissues were extracted and reverse transcribed into cDNA using a PrimeScript RT Reagent Kit (Takara, Otsu, Japan). β-actin or GAPDH was used as a reference control to normalize the gene expression values. Gene expression was analyzed using SYBR Green Master mix. The following primer sequences were used: mouse GITRL, forward 5′-CAAGTCCTCAAAGGGCAGAG-3′, reverse 5′-GGCAGTTGGCTTGAGTGAA-3′; mouse Foxp3, forward 5′-AAGAATGCCATCCGCCACAACC-3′, reverse 5′-GGCGTTGGCTCCTCTTCTTGC-3′; mouse GATA3, forward 5′-ATTACCACCTATCCGCCCTAT-3′, reverse 5′-CGGTTCTGCCCATTCATTTTAT-3′; mouse RORγt, forward 5′-ACAAATTGAAGTGATCCCTTGC-3′, reverse 5′-GGAGTAGGCCACATTACACTG-3′; mouse T-bet, forward 5′-AAGTTCAACCAGCACCAGACAGAG-3′, reverse 5′-GCCACGGTGAAGGACAGGAATG-3′; mouse β-actin, forward 5′-GTGCTATGTTGCTCTAGACTTCG-3′, reverse 5′-ATGCCACAGGATTCCATACC-3′; human GITRL, forward 5′-GTGAATAAGGTGTCTGACTGGA-3′, reverse 5′-GTCCCCAACATGCAATTCATAA-3′; and human GAPDH: forward 5′-CAGCGACACCCACTCCTCCACCTT-3′, reverse 5′-CATGAGGTCCACCACCCTGTTGCT-3′. The relative expression of genes was calculated using the 2-∆∆Ct analysis method.

### Statistical analysis

Data were analyzed using Graph Pad Prism version 5.0. Statistical comparisons among groups were performed using Student’s t-test, one-way ANOVA or two-way ANOVA with the Bonferroni post hoc test. Data are presented as the mean ± SEM, and a P value < 0.05 was considered significant.

## Results

### GITRL expression is increased on lung DCs from HDM-induced asthmatic mice

**Fig. 1:**
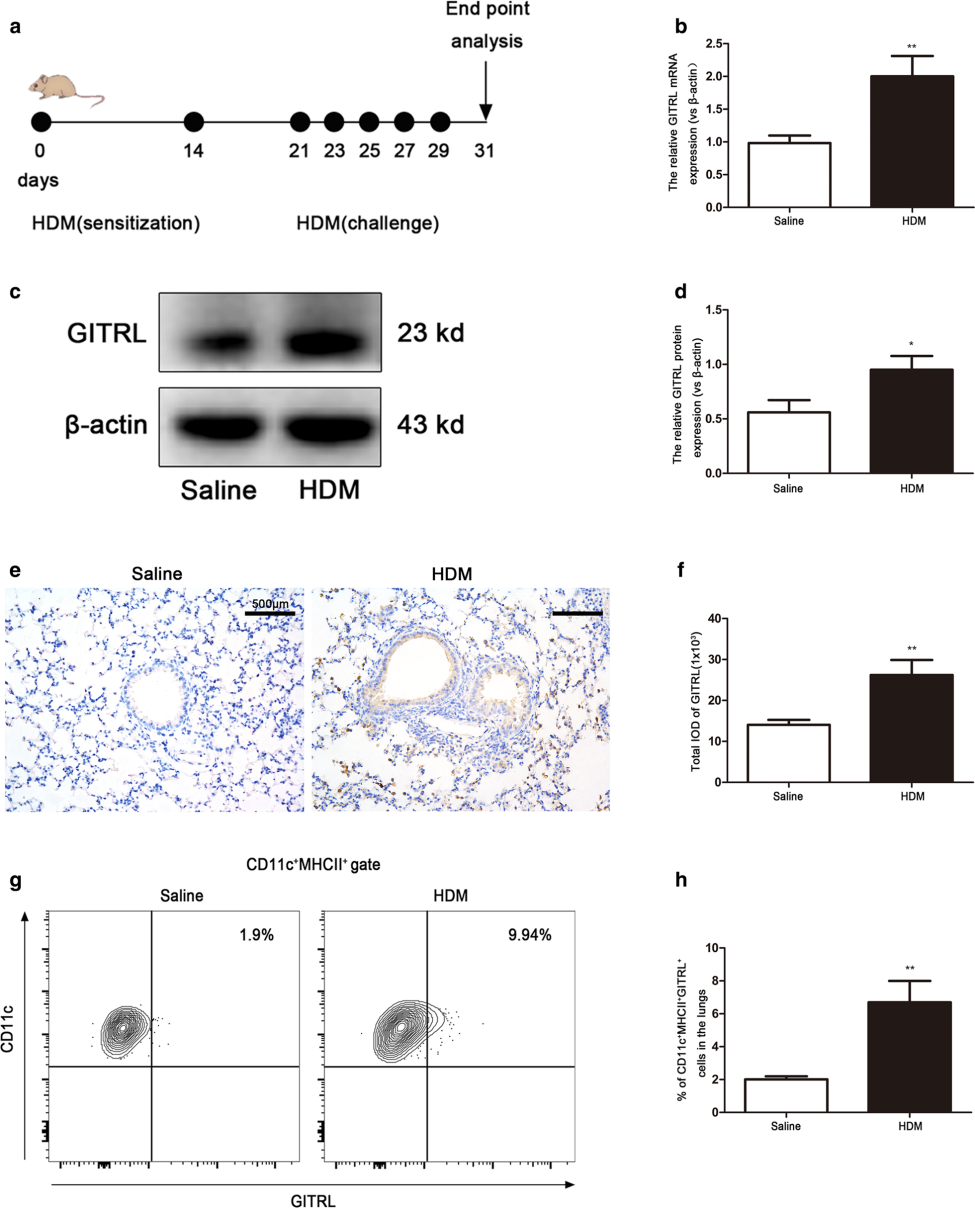
Increased GITRL expression on lung dendritic cells from HDM-induced asthmatic mice. **a** Mice were sensitized by intranasal house dust mite (HDM) administration (20 μg) on day 0 and day 14 and challenged by intranasal HDM (20 μg) administration on days 21, 23, 25, 27 and 29 before sacrifice on day 31. **b** GITRL mRNA expression in the lung tissues of normal saline and HDM-challenged mice. **c** Representative western blots of GITRL protein in the lung tissues of normal saline and HDM-challenged mice. **d** Quantification of western blots data from (**c**) (n = 8 per group). **e** Immunohistochemical staining for GITRL in the lung tissues of normal saline and HDM-challenged mice (× 200 magnification, scale bars = 500 μm). **f** The total integral optical density (IOD) for GITRL (n = 9 per group). **g** Flow cytometry analysis of the percentage of CD11c^+^MHCII^+^GITRL^+^ cells in the lung tissues of normal saline and HDM-challenged mice. **h** Statistical analysis of (**g**) (n = 8 per group). *P < 0.05, **P < 0.01

We used a murine model to assess whether sensitization and challenge with HDM modifies the cell surface expression of GITRL on lung DCs. The asthma modeling method used in this study is illustrated in Fig. 1a. Quantitative RT-PCR (Fig. 1b), western blot (Fig. 1c, d) and immunohistochemical (Fig. 1e, f) analyses confirmed that the level of GITRL in the lung was increased significantly in HDM-induced asthmatic mice compared with those in the normal saline group (P < 0.05). Furthermore, immunohistochemistry revealed strong expression of GITRL in cells close to the alveolar region. This was consistent with the normal location of DCs and alveolar macrophages in the lung. Furthermore, we found that the percentage of CD11c^+^MHCII^+^GITRL^+^ DCs in the lung increased significantly after HDM sensitization and challenge (Fig. 1g, h, P < 0.01).

### Allergen-induced airway inflammation and AHR are reduced in HDM-challenged GITRL knockdown mice

**Fig. 2:**
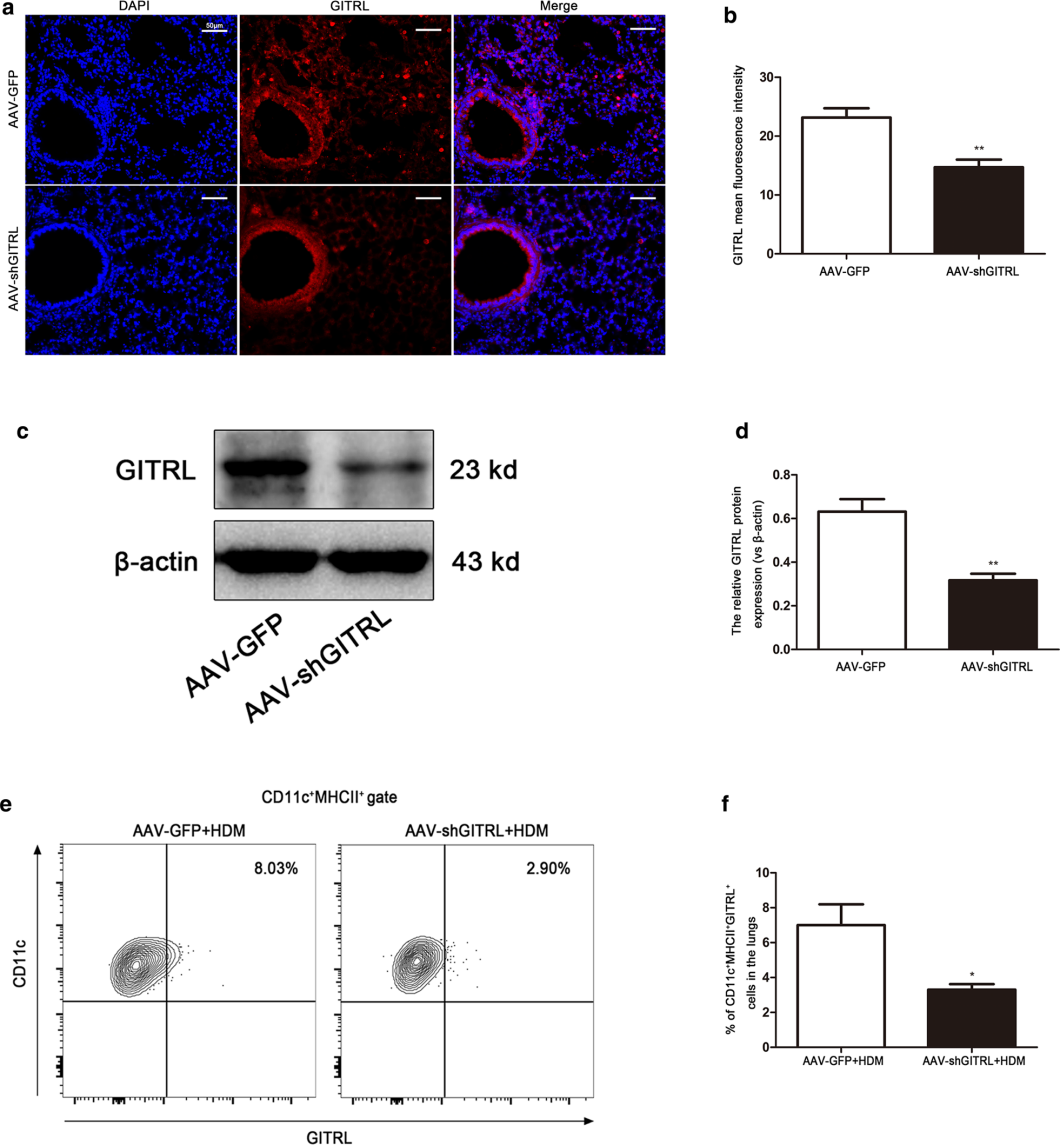
In vivo GITRL is successfully knocked down by intranasal delivery of the AAV vector. **a** Immunofluorescent staining for GITRL in AAV-GFP- and AAV-shGITRL-transduced mice (× 200 magnification, scale bars = 50 μm). **b** Quantification of the GITRL mean fluorescent intensity (MFI) in the lung (n = 5 per group). **c** Representative western blots of GITRL protein in the lungs of AAV-GFP- and AAV-shGITRL-transduced mice. **d** Quantification of western blot data from (**c**) (n = 5 per group). **e** Flow cytometry analysis of the percentage of CD11c^+^MHCII^+^GITRL^+^ cells in the lung tissues from HDM-challenged AAV-GFP and AAV-shGITRL mice. **f** Statistical analysis of (**e**) (n = 4 per group). *P < 0.05, **P < 0.01

**Fig. 3:**
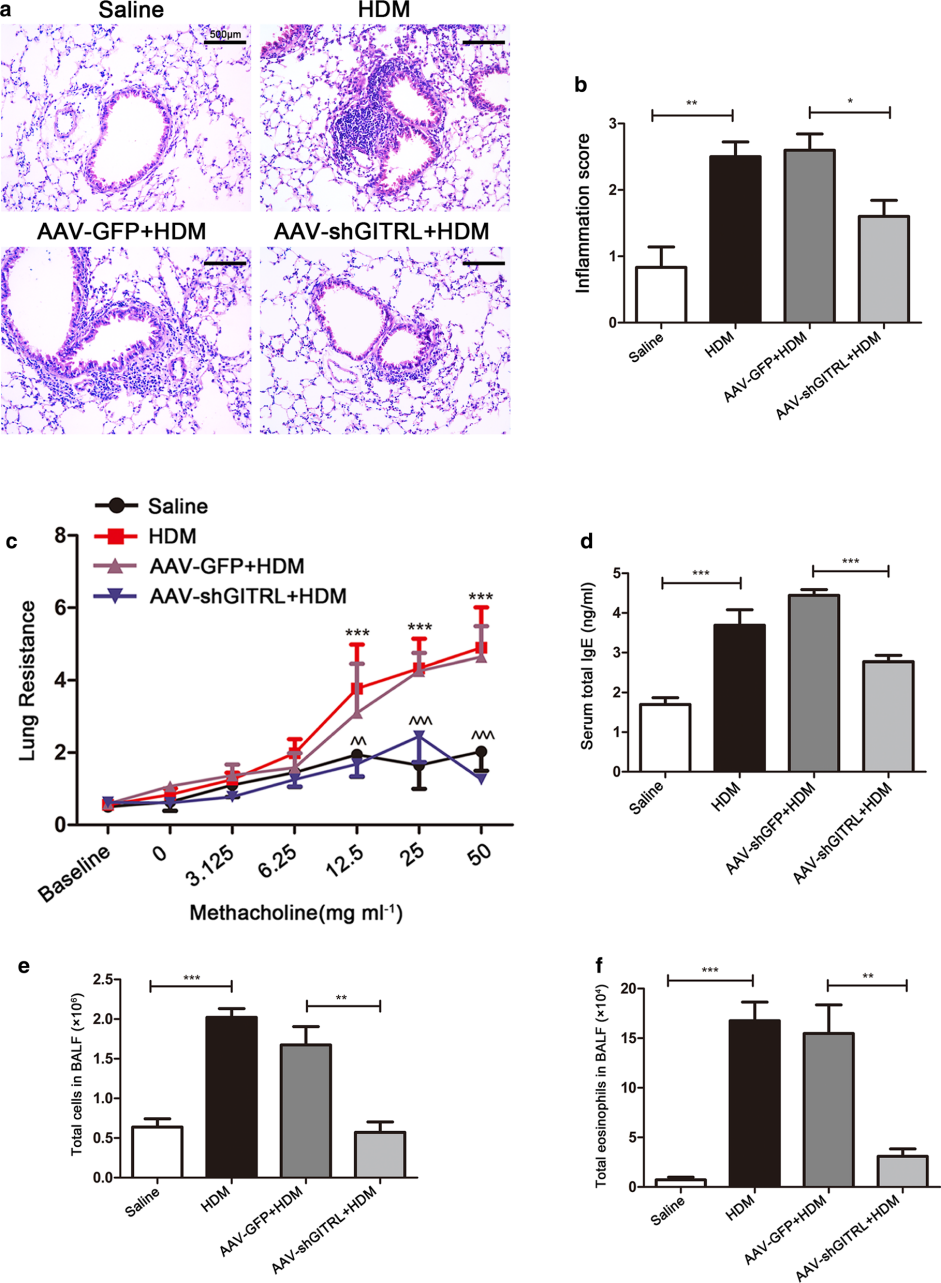
AAV-mediated GITRL knockdown reduces allergen-induced airway inflammation and airway hyperresponsiveness. **a** Representative hematoxylin and eosin (H&E) staining of lung sections in each group (× 200 magnification, scale bars = 500 μm). **b** Inflammation score in the airways and lungs (n = 5–6 per group). **c** Lung resistance to inhaled methacholine in each group (n = 4–6 per group). **d** Serum total IgE levels (n = 6–8 per group). **e** Total cells in bronchoalveolar lavage fluid (BALF) (n = 4–8 per group). **f** Total eosinophils in BALF (n = 4–8 per group). *P < 0.05, **P < 0.01, ***P < 0.001, HDM group versus Saline group; ^^P < 0.01, ^^^P < 0.001, HDM-challenged AAV-shGITRL group versus HDM-challenged AAV-GFP group

Having shown that GITRL is increased in asthmatic mice, experiments were next conducted to verify the role of GITRL in HDM-induced allergic asthma. To address this, we knocked down GITRL in the lung using AAV-shGITRL. Immunofluorescence (Fig. 2a, b) and western blot (Fig. 2c, d) demonstrated that the GITRL expression was significantly knocked down in the lung (P < 0.01). Flow cytometry analyses showed that GITRL on the surface of DCs was successfully knocked down after HDM sensitization and challenge (Fig. 2e, f, P < 0.05). We next examined the effects of GITRL knockdown on airway inflammation and AHR. As shown in Fig. 3a–f, HDM-challenged AAV-shGITRL mice had less inflammatory cells infiltration around the bronchus than AAV-GFP mice, and the total number of BALF cells, eosinophils and serum total IgE were significantly reduced (P < 0.05). Furthermore, lung resistance was decreased in HDM-challenged AAV-shGITRL mice compared with AAV-GFP mice (Fig. 3c, P < 0.01). Collectively, these data demonstrate that HDM-challenged AAV-shGITRL mice have a phenotype of decreased allergic airway inflammation and AHR.

### The balance of Th1/Th2 and Th17/Treg cells is partially restored in HDM-challenged GITRL knockdown mice

**Fig. 4:**
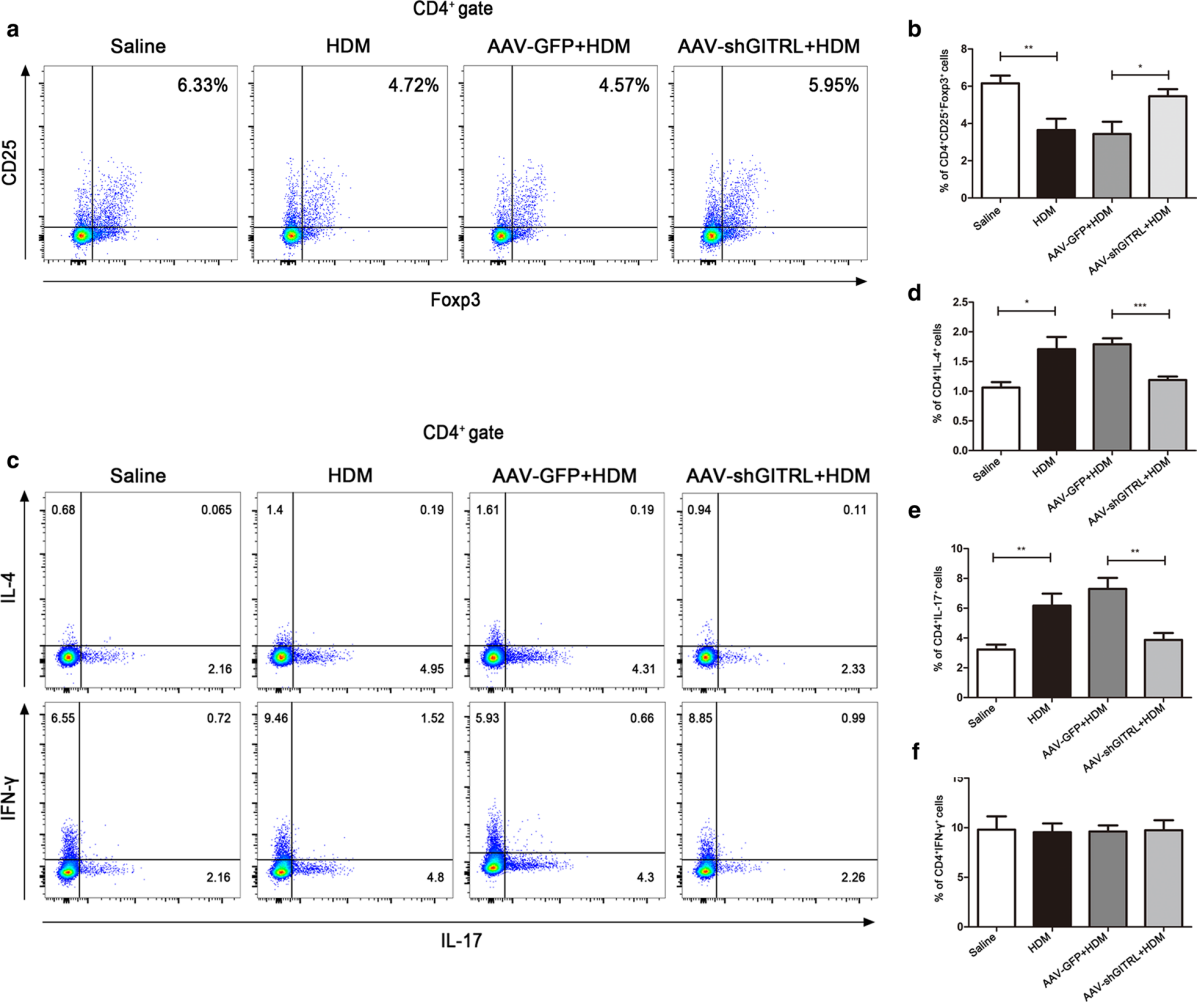
The balance of Th1/Th2 and Th17/Treg cells is partially restored in HDM-challenged GITRL knockdown mice. **a** Flow cytometry analysis of the percentage of CD4^+^CD25^+^Foxp3^+^ T cells (Tregs) in the lung tissues of mice. **b** Statistical analysis of (**a**) (n = 4–6 per group). **c** Flow cytometry analysis of the percentage of CD4^+^IL-4^+^ T cells (Th2), CD4^+^IFN-γ^+^ T cells (Th1) and CD4^+^IL-17^+^ T cells (Th17) in the lung tissues of mice. **d**–**f** Statistical analysis of (**c**) (n = 5 per group). *P < 0.05, **P < 0.01, ***P < 0.001

In view of the role of GITRL expressed on DCs in CD4^+^ T cell differentiation, we asked whether GITRL could impact the differentiation of CD4^+^ T cells in asthma. Flow cytometry demonstrated a significant reduction in the percentage of CD4^+^CD25^+^Foxp3^+^ T cells (Tregs) in the lung of HDM-challenged mice compared with normal saline mice (Fig. 4a, b, P < 0.01). However, the percentage of Tregs was upregulated in the lungs of HDM-challenged AAV-shGITRL mice compared with HDM-challenged AAV-GFP mice (Fig. 4a, b, p < 0.05). In addition, the percentage of Th2 and Th17 cells in HDM-challenged mice was increased compared with normal saline mice (Fig. 4c–e, P < 0.05). Compared with HDM-challenged AAV-GFP mice, GITRL knockdown markedly decreased the percentage of Th2 and Th17 cells under HDM challenge (Fig. 4c–e, P < 0.01). There was no difference in the percentage of Th1 cells in each group (Fig. 4c, f, P > 0.05). Thus, these data suggest that the upregulated expression of GITRL by lung DCs might in part contribute to the Th1/Th2 and Th17/Treg cell imbalance in HDM-challenged mice.

### Cell surface GITRL expression is increased on mouse BMDCs stimulated by HDM in vitro

**Fig. 5:**
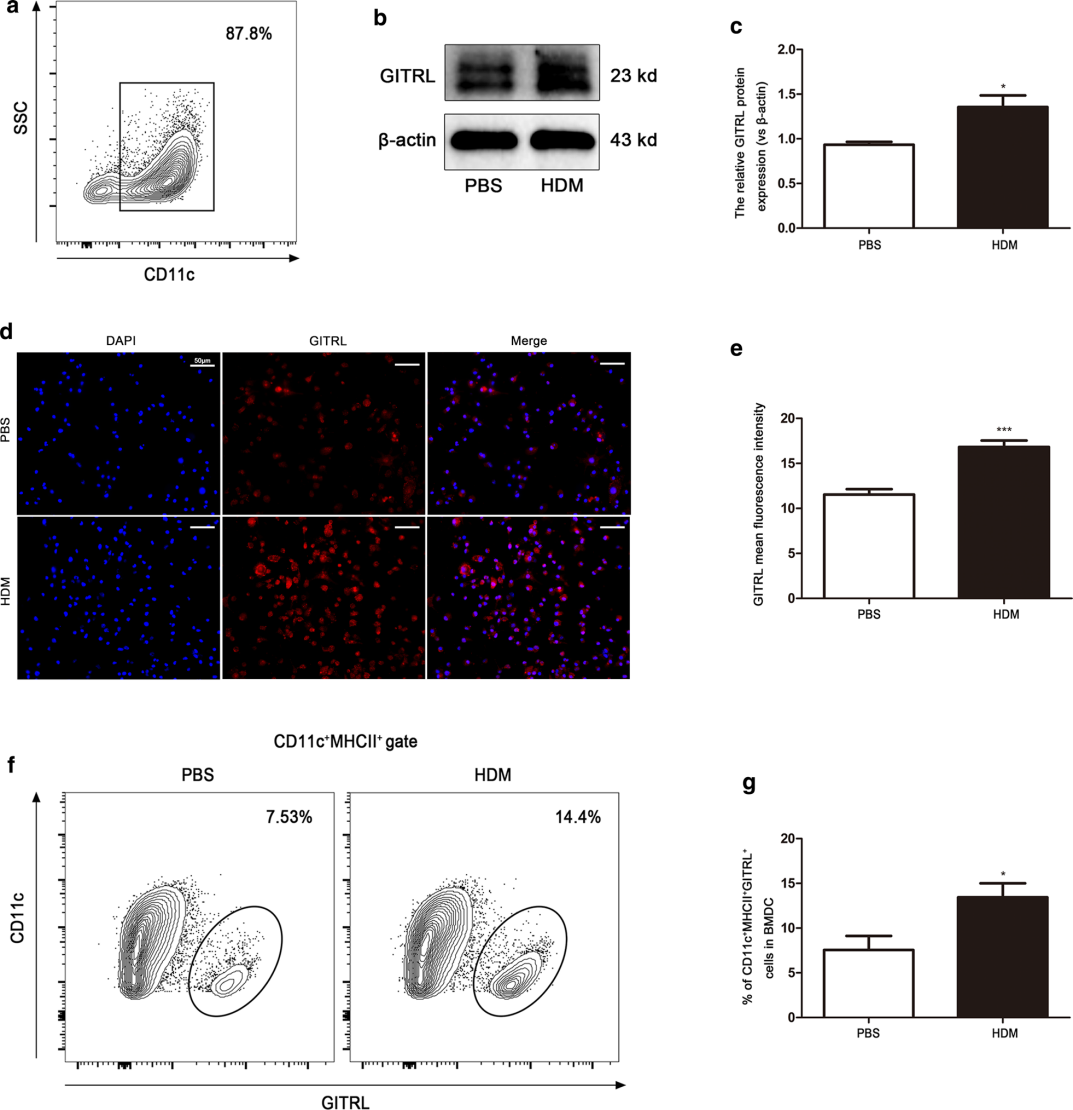
GITRL is increased on mouse bone marrow-derived dendritic cells stimulated by HDM in vitro. **a** Flow cytometry analysis of the percentage of CD11c^+^ cells. **b** Representative western blots of GITRL protein in PBS or HDM-treated BMDCs. **c** Quantification of western blots data from (**b**) (n = 4–5 per group). **d** Immunofluorescent staining for GITRL in PBS or HDM-treated BMDCs (× 200 magnification, scale bars = 50 μm). **e** Quantification of the GITRL mean fluorescent intensity (MFI) in PBS- or HDM-treated BMDCs (n = 6 per group). **f** Flow cytometry analysis of the percentage of CD11c^+^MHCII^+^GITRL^+^ cells in PBS- or HDM-treated BMDCs. **g** Statistical analysis of (**f**) (n = 4–5 per group). *P < 0.05, ***P < 0.001

In vitro experiments were conducted to further verify the role of GITRL on the DC surface in HDM-induced asthma. BMDCs were extracted from mice and stimulated with HDM. More than 80% of the BMDCs expressed CD11c as determined by flow cytometry (Fig. 5a). After HDM stimulation, western blot (Fig. 5b, c), immunofluorescence (Fig. 5d, e) and flow cytometry (Fig. 5f, g) showed that GITRL on BMDCs increased (P < 0.05), which is consistent with the results of the animal model.

### The balance of Th1/Th2 and Th17/Treg cells is partially restored by knocking down GITRL on BMDCs cocultured with splenocytes under HDM stimulation

**Fig. 6:**
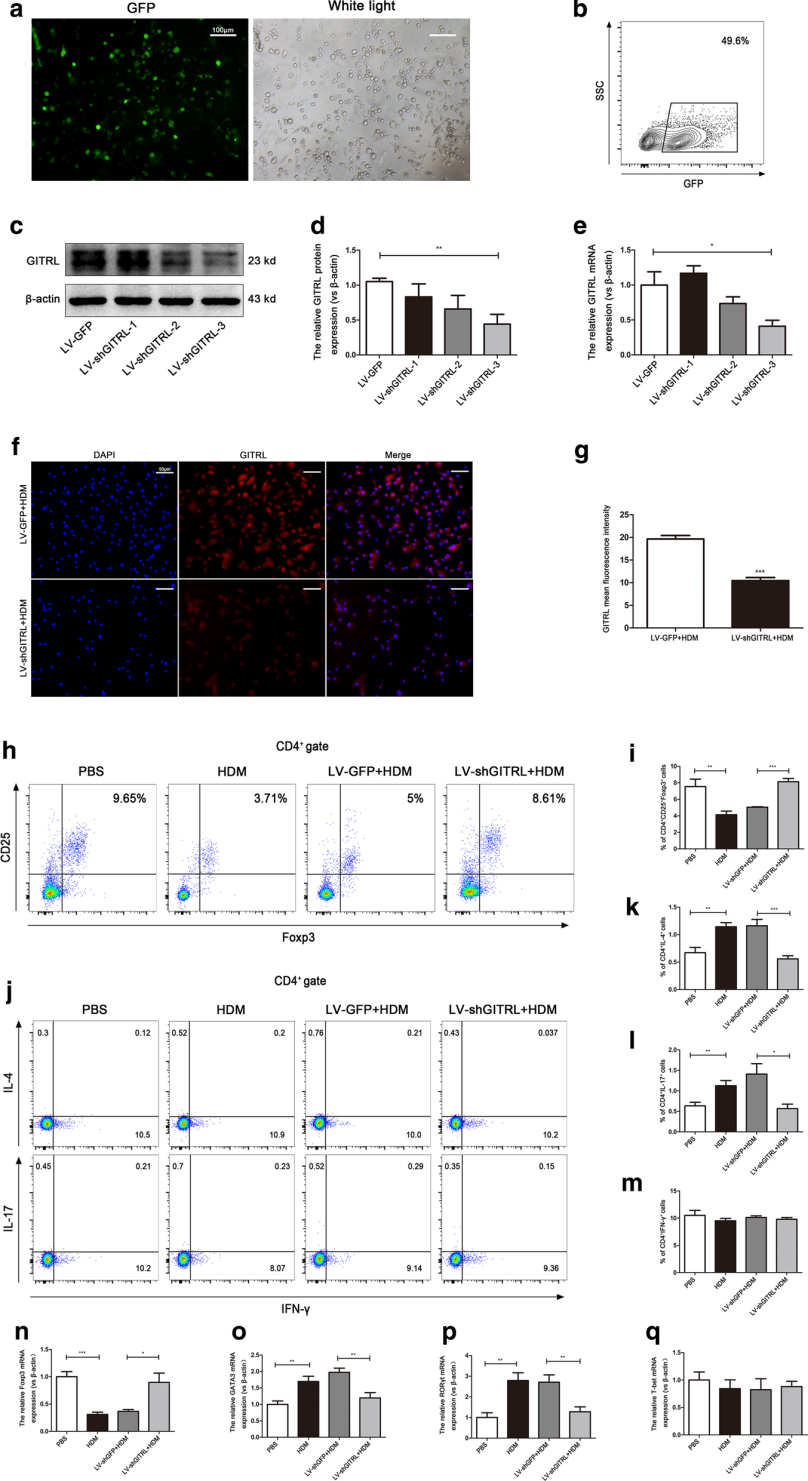
Knockdown of GITRL on BMDCs partially restored the balance of Th1/Th2 and Th17/Treg cells. **a** Expression of GFP and the morphologic characteristics of BMDCs were observed under a fluorescence microscope (× 100 magnification, scale bars = 100 μm). **b** Flow cytometry analysis of the percentage of GFP^+^ cells in BMDCs. **c** Representative western blots of GITRL protein in LV-GFP-, LV-shGITRL-1-, LV-shGITRL-2-, and LV-shGITRL-3-transduced BMDCs. **d** Quantification of western blot data from (**c**) (n = 4—5 per group). **e** GITRL mRNA expression in each group (n = 6—7 per group). **f** Immunofluorescent staining for GITRL in HDM-treated LV-GFP and LV-shGITRL BMDCs (× 200 magnification, scale bars = 50 μm). **g** Quantification of the GITRL mean fluorescent intensity (MFI) in HDM treated LV-GFP and LV-shGITRL BMDCs (n = 6 per group). **h** Flow cytometry analysis of the percentage of CD4^+^CD25^+^Foxp3^+^ T cells (Tregs) in splenocytes cocultured with BMDCs. **i** Statistical analysis of (**h**) (n = 6 per group). **j** Flow cytometry analysis of the percentage of CD4^+^IL-4^+^ T cells (Th2), CD4^+^IFN-γ^+^ T cells (Th1) and CD4^+^IL-17^+^ T cells (Th17) in splenocytes cocultured with BMDCs. **k**–**m** Statistical analysis of (**j**) (n = 6 per group). **n**–**q** Foxp3, GATA3, RORγt and T-bet mRNA expression in each group (n = 6 per group). *P < 0.05, **P < 0.01, ***P < 0.001

To further explore the role of GITRL in CD4^+^ T cell differentiation in vitro, we knocked down GITRL on BMDCs using LV-shGITRL. GFP was visualized by fluorescence microscopy (Fig. 6a). The percentage of GFP-positive cells was approximately 50% by flow cytometry (Fig. 6b). Subsequently, we tested the efficacy of GITRL shRNA transfection in downregulating GITRL by western blot. As shown in Fig. 6c–e, LV-shGITRL-3 had the best knockdown effect on GITRL, which was approximately 50% lower (P < 0.05). Therefore, LV-shGITRL-3 was used for our experiment. Furthermore, immunofluorescence showed that after HDM stimulation, GITRL on the surface of LV-shGITRL BMDCs decreased compared with that on the surface of LV-GFP BMDCs (Fig. 6f, g, P < 0.01). Next, we examined the effect of knocking down GITRL on the surface of BMDCs on the differentiation of CD4^+^ T cells. BMDCs were cocultured with splenocytes with HDM stimulation. Then, the cells were analyzed by flow cytometry for CD4^+^ T cell subsets. Flow cytometry demonstrated a significant reduction in the percentage of Tregs in cells treated with HDM compared with PBS-treated cells (Fig. 6h, i, P < 0.01). However, under HDM stimulation, the percentage of Tregs with GITRL knockdown was markedly increased compared with the LV-GFP group (Fig. 6h, i, P < 0.001). In addition, the percentages of Th2 and Th17 cells in HDM-treated cells were increased compared with those in PBS-treated cells (Fig. 6j–l, P < 0.01). Compared with the LV-GFP group, the percentages of Th2 and Th17 cells were markedly decreased with GITRL knockdown under HDM stimulation (Fig. 6j–l, P < 0.05). There was no difference in the percentage of Th1 cells in each group (Fig. 6j, m, P > 0.05). These results were consistent with the results of the in vivo experiments. We next addressed whether GITRL knockdown could affect the amount of Foxp3, GATA3, RORγt and T-bet in cocultured cells. Quantitative RT-PCR showed that Foxp3 expression decreased significantly, while GATA3 and RORγt mRNA increased in cells treated with HDM compared with PBS-treated cells (Fig. 6n–p, P < 0.01). After GITRL knockdown, Foxp3 mRNA expression increased, while GATA3 and RORγt decreased (Fig. 6n–p, P < 0.05). However, there was no significant difference in T-bet mRNA expression in each group (Fig. 6q, P > 0.05). Collectively, these experiments further show that GITRL on DCs might modulate the differentiation of CD4^+^ T cells and in part contribute to the imbalance of Th1/Th2 and Th17/Treg cells.

### GITRL stimulation inhibits Treg differentiation and promotes Th2 and Th17 cell differentiation

**Fig. 7:**
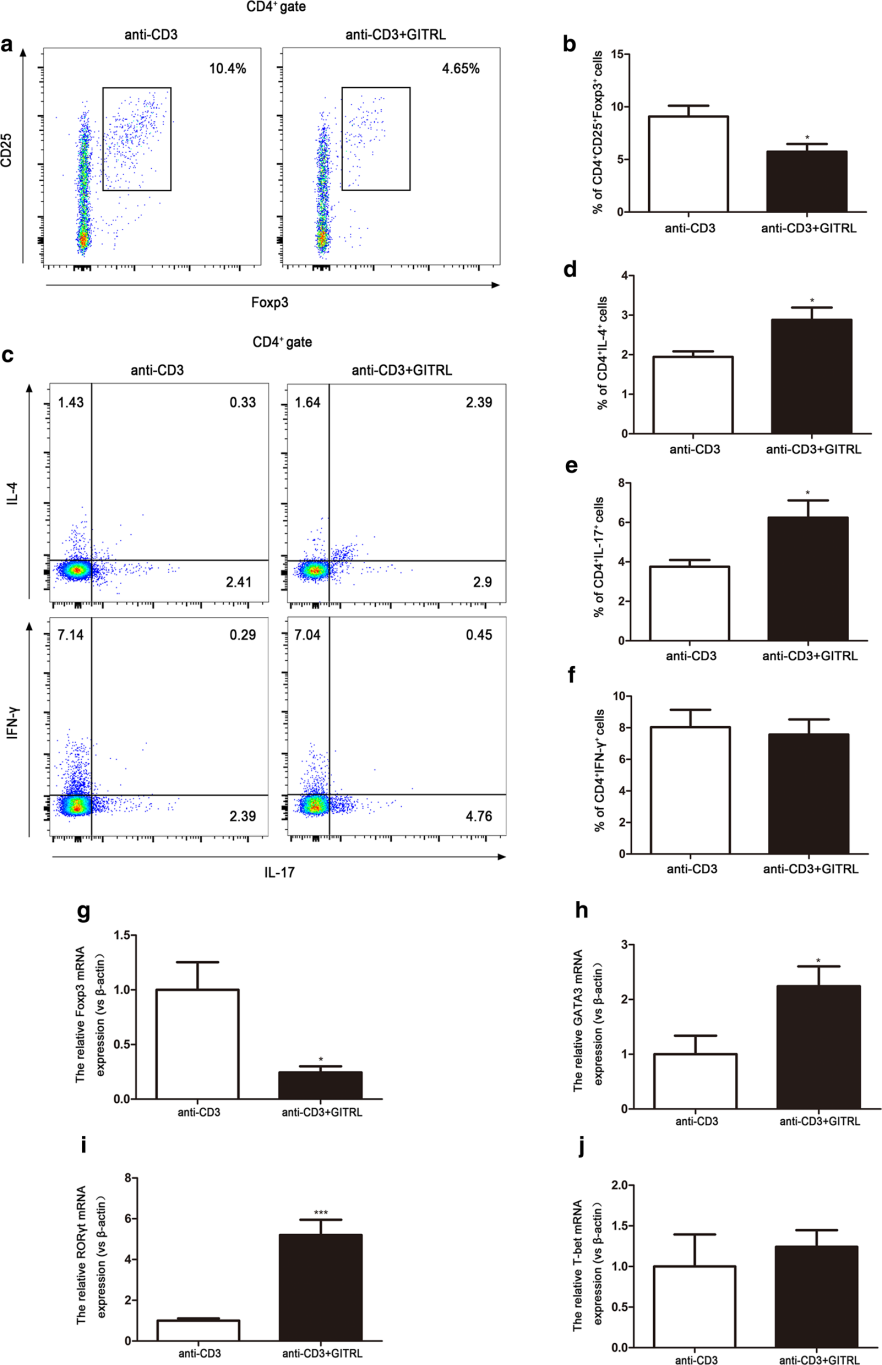
GITRL stimulation inhibits Treg cell differentiation and promotes Th2 and Th17 cell differentiation. **a** Flow cytometry analysis of the percentage of CD4^+^CD25^+^Foxp3^+^ T cells (Tregs) in splenocytes stimulated with anti-CD3 or anti-CD3 plus GITRL. **b** Statistical analysis of (**a**) (n = 5 per group). **c** Flow cytometry analysis of the percentage of CD4^+^IL-4^+^ T cells (Th2), CD4^+^IFN-γ^+^ T cells (Th1) and CD4^+^IL-17^+^ T cells (Th17) in splenocytes stimulated with anti-CD3 or anti-CD3 plus GITRL. **d**–**f** Statistical analysis of (**c**) (n = 5 per group). **g**–**j** Foxp3, GATA3, RORγt and T-bet mRNA expression in each group (n = 5 per group). *P < 0.05, ***P < 0.001

To determine whether CD4 + T cell differentiation was affected directly by GITRL, we stimulated murine splenocytes with anti-CD3 alone or anti-CD3 plus GITRL. As shown in Fig. 7a, b, flow cytometry demonstrated a significant reduction in the percentage of Tregs in cells stimulated with anti-CD3 plus GITRL compared with cells treated with anti-CD3 alone (P < 0.05). Moreover, in the presence of GITRL, the percentages of Th2 and Th17 cells were increased (Fig. 7c–e, P < 0.05). There was no difference in the percentage of Th1 cells (Fig. 7c, f, P > 0.05). We next addressed whether GITRL stimulation could affect the amount of Foxp3, GATA3, RORγt and T-bet mRNA in splenocytes. Quantitative RT-PCR showed that Foxp3 mRNA expression decreased significantly, while GATA3 and RORγt mRNA expression increased after GITRL stimulation (Fig. 7g–i, P < 0.05). There was no significant difference in T-bet mRNA expression between the two groups (Fig. 7j, P > 0.05). These experiments further confirmed that GITRL may directly modulate the differentiation of CD4^+^ T cells, leading to the imbalance of Th1/Th2 and Th17/Treg cells.

### GITRL mRNA expression is increased in peripheral blood from asthmatic children

**Fig. 8:**
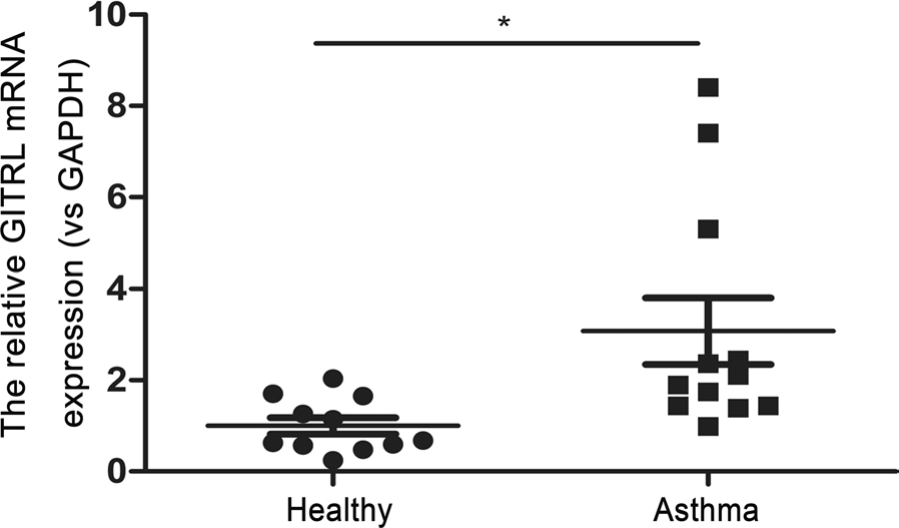
Increased expression of GITRL mRNA in asthmatic children. GITRL mRNA expression in the peripheral blood from healthy children (n = 11) and asthmatic children (n = 12). *P < 0.05

In view of the essential role of GITRL in allergy, combined with our results in animal model in vivo and in vitro, we further enrolled 12 children with asthma and eleven children without respiratory diseases or autoimmune diseases and then examined the expression of GITRL in peripheral blood. As we expected, quantitative RT-PCR showed that the GITRL mRNA level was higher in asthmatic children than in the healthy control group (Fig. 8, P < 0.05). Thus, GITRL is increased in asthmatic children.

## Discussion

DCs are considered professional APCs as a result of their essential roles in immune responses. Lung DCs are located throughout the respiratory tract and have significant roles in lung homeostasis and in the pathogenesis of pulmonary diseases. Lung DCs have become an important cellular therapeutic target of new vaccine strategies for a variety of human lung diseases, including asthma [28]. GITRL, a member of the tumor necrosis factor superfamily (TNFSF), is mainly expressed on DCs, B cells and macrophages [10, 11]. TNFSF members not only regulate cellular differentiation, survival, and programmed death but also play a critical role in the immune system [29]. Previous studies have shown that TNFSF members such as OX40L, TRAIL, and LIGHT play important roles in asthma [30–32]. However, limited data exist regarding the function of GITRL in allergen-mediated asthma. It was reported that GITRL was positively correlated with group 2 innate lymphocyte (ILC2)-associated molecules, thus exacerbating ovalbumin (OVA)-induced asthma [21]. Furthermore, mice treated with GITRL blockers or GITR-deficient mice exhibited reduced inflammatory symptoms in models of lung and gut inflammation, pancreatitis, diabetes, allograft rejection, spinal cord injury and other diseases [33–36]. These findings suggest that blocking the GITR–GITRL interaction may be suitable for the treatment of inflammatory diseases. Thus, in this study, we explored whether regulating GITRL expressed on lung DCs can prevent asthma and its mechanism of action.

Since HDM is the most common allergen implicated in human allergic asthma, we used HDM to establish an asthma mouse model. Similarly, we found that HDM stimulation promotes GITRL expression on the lung DCs of mice. This is similar to prior reports demonstrating the upregulation of cell surface GITRL expression on DCs in response to proinflammatory stimuli [37, 38]. It has been shown that AAV6 is one of the most efficient serotypes used for lung gene transfer [39, 40]. To confirm the role of GITRL in asthma, we established a mouse model of GITRL knockdown in the lung using AAV6 vectors to characterize the role of GITRL in regulating HDM-induced asthma. After GITRL on the lung DCs was successfully knocked down, we found that HDM-challenged AAV-shGITRL mice display decreased airway inflammation, serum total IgE levels and AHR. This suggests that GITRL, as a positive regulator, promotes airway inflammation and AHR in HDM-induced asthma. Knockdown of GITRL can effectively alleviate lung inflammation and AHR in asthmatic mice.

Furthermore, we sought to elucidate the mechanism by which GITRL aggravates asthma. It is known that the balance of Th1/Th2 and Th17/Treg cells plays a central role in the pathogenesis of allergic asthma. It was reported that GITR activation leads to an exacerbation of murine allergic airway inflammation with elevated production of OVA-specific Th1 and Th2 cytokines [22]. In addition, previous studies have shown that GITR/GITRL can abrogate the immunosuppressive functions of Tregs [13, 36]. Furthermore, treatment with soluble GITRL reduces the suppression of Tregs and restores the proliferation of CD4^+^CD25^−^ T cells in cancer [20]. In other studies, GITRL could aggravate autoimmune diseases by promoting Th17 cell expansion [41, 42]. This led us to conjecture that GITRL might promote the development of asthma by modulating the differentiation of CD4^+^ T cells. Therefore, we measured the proportion of Th1, Th2, Th17 and Treg cells and the cytokines secreted by Th1, Th2, Th17 and Treg cells in HDM-challenged AAV-shGITRL mice. Here, we showed that GITRL knockdown markedly decreased the percentages of Th2 and Th17 cells and increased the percentage of Tregs, while the percentage of Th1 cells did not change significantly. Our results are consistent with those of another study, which mentioned that GITR stimulation costimulates Th2 cytokine production but has no effect on Th1 cytokine production [23]. Although another study showed that recombinant mouse GITRL (rmGITRL) promoted the proliferation of Th1 cells at low concentrations, it did not at high concentrations [15]. However, the effect of rmGITRL on the proliferation of Th2 cells increased with the increase of rmGITRL concentration. Therefore, we did not observe the change of Th1 cells in our study, which may be caused by different intensity of antigen stimulation. This suggests that GITRL may mainly affect Treg, Th2 and Th17 cells. Thus, GITRL may promote the development of asthma by modulating the differentiation of CD4^+^ T cells and disturbing the balance of Th1/Th2 and Th17/Treg cells.

Since we failed to specifically knock down GITRL on DCs in vivo, we conducted in vitro experiments to confirm the role of GITRL in the differentiation of CD4^+^ T cells. First, we cultured BMDCs and stimulated them with HDM. We found that GITRL expression on BMDCs was upregulated after HDM stimulation, which is consistent with our findings in animal model. Then, we knocked down GITRL on BMDCs with LV-shGITRL and coculture BMDCs with splenocytes to characterize the role of GITRL expression in the balance of Th1/Th2 and Th17/Treg cells. We showed that Th1/Th2 and Th17/Treg cells balances are partially restored by knocking down GITRL on BMDCs. Since Foxp3, GATA3, RORγt and T-bet are the “master regulator” transcription factors for Treg, Th2, Th17 and Th1 cell differentiation, respectively, we tested the impact of GITRL on them. We showed that Foxp3 increased, while GATA3 and RORγt expression decreased after knocking down GITRL on BMDCs. To further determine whether CD4^+^ T cells differentiation was affected directly by GITRL, we stimulated splenocytes with GITRL protein in vitro. Interestingly, we found that after stimulating splenocytes with GITRL, the proportion of Tregs decreased, and the proportion of Th2 and Th17 cells increased, accompanied by a decrease in Foxp3 and an increase in GATA3 and RORγt. Collectively, these results confirm the conclusion that GITRL on DCs may promote asthma by modulating CD4^+^ T cell differentiation and disturbing the balance of Th1/Th2 and Th17/Treg cells. In the future, we can use mixed bone marrow chimera to detect which CD4^+^ T cell subsets are directly mediated by GITRL in a cell-intrinsic manner, and study the effect of GITRL on the apoptosis or proliferation of different CD4^+^ T cell subsets.

To determine whether the above findings are related to human beings, we used quantitative RT-PCR to measure the expression of GITRL mRNA in the peripheral blood of asthmatic children. We found that asthmatic children have increased GITRL mRNA expression compared with nonasthmatic subjects. This change is consistent with our results in animal experiments and in vitro experiments, which further indicates the importance of GITRL in the pathogenesis of asthma and may provide new ways of clinical treatment. Many treatment approaches targeting TNFSF/TNFRSF members have been applied to various cancers. Agents targeting GITR are currently under investigation in clinical trials. It has been found that MK-4166, a human GITR agonist, can reduce the expression of Foxp3 mRNA in human tumor infiltrating Tregs [43]. The safety and tolerability of MK-4166 antibody in the treatment of advanced solid tumors are currently being evaluated in a phase I study (NCT02132754) [43]. Moreover, a recent study screened drugs that can inhibit the binding of GITR and GITRL from nearly 10,000 drugs approved by FDA. It was found that minocycline can inhibit the proliferation of T lymphocytes stimulated by GITRL and regulate the expression of IL-17 and RORγt in T lymphocytes [44]. Moreover, other studies have found that minocycline can reduce the symptoms of asthma [45, 46]. As blocking GITRL showed a protective effect for asthma in our research and other research on GITRL-associated clinical trials and blocking or agonist drugs of GITR are relatively mature, GITRL may be a therapeutic target for asthma in the future, which has important clinical significance. Although the main concern about the use of GITRL inhibitors is that theoretically GITRL inhibition may be beneficial to the development of immunosuppression and infectious diseases [34], further clinical trials are needed to confirm this. GITRL inhibitors still have broad application prospects in the treatment of allergic diseases and autoimmune diseases. We should balance the benefits and risks of GITRL inhibitors.

## Conclusions

In summary, the present study for the first time demonstrated a role for GITRL on the surface of DCs in the pathogenesis of immune responses in experimental HDM-induced asthma. We demonstrated that GITRL promotes allergic airway inflammation and AHR by modulating CD4^+^ T cell differentiation and disturbing the balance of Th1/Th2 and Th17/Treg cells. Furthermore, we found that GITRL expression is increased in peripheral blood from asthmatics, which suggests a possible role for GITRL in the development of asthma in humans. This study further suggests that GITRL inhibitors may be a potential immunotherapy for asthma, which provides new insights for asthma treatment.

## Data Availability

All data generated or analysed during this study are included in this published article. The datasets used and/or analysed during the current study are available from the corresponding author on reasonable request.

## Abbreviations

GITRL: Glucocorticoid-induced tumor necrosis factor receptor family-related protein ligand
DCs: Dendritic cells
HDM: House dust mite
BMDCs: Bone marrow dendritic cells
AHR: Airway hyperresponsiveness
Tregs: Regulatory T cells
Th2: T helper 2 cells
APCs: Antigen-presenting cells
GITR: Glucocorticoid-induced tumor necrosis factor receptor-related receptor
IHC: Immunohistochemical
IOD: Integral optical density
BALF: Bronchoalveolar lavage fluid
BM: Bone marrow
LV: Lentiviral vectors
AAV: Adeno-associated virus
GFP: Green fluorescent protein
TNFSF: Tumor necrosis factor superfamily

## References

[1] Collaborators GBDCRD (2017). Global, regional, and national deaths, prevalence, disability-adjusted life years, and years lived with disability for chronic obstructive pulmonary disease and asthma, 1990–2015: a systematic analysis for the Global Burden of Disease Study 2015. Lancet Respir Med.

[2] Tortola L, Pawelski H, Sonar SS, Ampenberger F, Kurrer M, Kopf M (2019). IL-21 promotes allergic airway inflammation by driving apoptosis of FoxP3(+) regulatory T cells. J Allergy Clin Immunol.

[3] Lambrecht BN, Hammad H, Fahy JV (2019). The cytokines of asthma. Immunity.

[4] Tumes DJ, Papadopoulos M, Endo Y, Onodera A, Hirahara K, Nakayama T (2017). Epigenetic regulation of T-helper cell differentiation, memory, and plasticity in allergic asthma. Immunol Rev.

[5] Vroman H, Bergen IM, van Hulst JAC, van Nimwegen M, van Uden D, Schuijs MJ, Pillai SY, van Loo G, Hammad H, Lambrecht BN (2018). TNF-α-induced protein 3 levels in lung dendritic cells instruct T(H)2 or T(H)17 cell differentiation in eosinophilic or neutrophilic asthma. J Allergy Clin Immunol.

[6] Noval Rivas M, Chatila TA (2016). Regulatory T cells in allergic diseases. J Allergy Clin Immunol.

[7] Deckers J, De Bosscher K, Lambrecht BN, Hammad H (2017). Interplay between barrier epithelial cells and dendritic cells in allergic sensitization through the lung and the skin. Immunol Rev.

[8] Vroman H, van den Blink B, Kool M (2015). Mode of dendritic cell activation: the decisive hand in Th2/Th17 cell differentiation. Implications in asthma severity?. Immunobiology.

[9] Nocentini G, Giunchi L, Ronchetti S, Krausz LT, Bartoli A, Moraca R, Migliorati G, Riccardi C (1997). A new member of the tumor necrosis factor/nerve growth factor receptor family inhibits T cell receptor-induced apoptosis. Proc Natl Acad Sci U S A.

[10] Chattopadhyay K, Ramagopal U, Brenowitz M, Nathenson S, Almo S (2008). Evolution of GITRL immune function: murine GITRL exhibits unique structural and biochemical properties within the TNF superfamily. Proc Natl Acad Sci U S A.

[11] Yu KY, Kim HS, Song SY, Min SS, Jeong JJ, Youn BS (2003). Identification of a ligand for glucocorticoid-induced tumor necrosis factor receptor constitutively expressed in dendritic cells. Biochem Biophys Res Commun.

[12] Clouthier DL, Watts TH (2014). Cell-specific and context-dependent effects of GITR in cancer, autoimmunity, and infection. Cytokine Growth Factor Rev.

[13] Stephens GL, McHugh RS, Whitters MJ, Young DA, Luxenberg D, Carreno BM, Collins M, Shevach EM (2004). Engagement of glucocorticoid-induced TNFR family-related receptor on effector T cells by its ligand mediates resistance to suppression by CD4+CD25+ T cells. J Immunol.

[14] Ronchetti S, Nocentini G, Riccardi C, Pandolfi PP (2002). Role of GITR in activation response of T lymphocytes. Blood.

[15] Tone M, Tone Y, Adams E, Yates SF, Frewin MR, Cobbold SP, Waldmann H (2003). Mouse glucocorticoid-induced tumor necrosis factor receptor ligand is costimulatory for T cells. Proc Natl Acad Sci U S A.

[16] Watts TH (2005). TNF/TNFR family members in costimulation of T cell responses. Annu Rev Immunol.

[17] Esparza EM, Arch RH (2006). Signaling triggered by glucocorticoid-induced tumor necrosis factor receptor family-related gene: regulation at the interface between regulatory T cells and immune effector cells. Front Biosci.

[18] Joetham A, Ohnishi H, Okamoto M, Takeda K, Schedel M, Domenico J, Dakhama A, Gelfand EW (2012). Loss of T regulatory cell suppression following signaling through glucocorticoid-induced tumor necrosis receptor (GITR) is dependent on c-Jun N-terminal kinase activation. J Biol Chem.

[19] Ma J, Wang S, Ma B, Mao C, Tong J, Yang M, Wu C, Jiao Z, Lu L, Xu H (2011). Dendritic cells engineered to express GITRL enhance therapeutic immunity in murine Lewis lung carcinoma. Cancer Lett.

[20] Pedroza-Gonzalez A, Verhoef C, Ijzermans JN, Peppelenbosch MP, Kwekkeboom J, Verheij J, Janssen HL, Sprengers D (2013). Activated tumor-infiltrating CD4+ regulatory T cells restrain antitumor immunity in patients with primary or metastatic liver cancer. Hepatology.

[21] Zhang M, Wan J, Xu Y, Zhang D, Peng J, Qi C, Guo Q, Xia S, Su Z, Wang S, Xu H (2017). Simultaneously increased expression of glucocorticoidinduced tumor necrosis factor receptor and its ligand contributes to increased interleukin5/13producing group 2 innate lymphocytes in murine asthma. Mol Med Rep.

[22] Patel M, Xu D, Kewin P, Choo-Kang B, McSharry C, Thomson NC, Liew FY (2005). Glucocorticoid-induced TNFR family-related protein (GITR) activation exacerbates murine asthma and collagen-induced arthritis. Eur J Immunol.

[23] Motta AC, Vissers JL, Gras R, Van Esch BC, Van Oosterhout AJ, Nawijn MC (2009). GITR signaling potentiates airway hyperresponsiveness by enhancing Th2 cell activity in a mouse model of asthma. Respir Res.

[24] Halbert CL, Miller AD (2004). AAV-mediated gene transfer to mouse lungs. Methods Mol Biol.

[25] van Lieshout LP, Domm JM, Wootton SK (2019). AAV-mediated gene delivery to the lung. Methods Mol Biol.

[26] Lutz MB, Kukutsch N, Ogilvie AL, Rossner S, Koch F, Romani N, Schuler G (1999). An advanced culture method for generating large quantities of highly pure dendritic cells from mouse bone marrow. J Immunol Methods.

[27] Zhou Z, Chen H, Xie R, Wang H, Li S, Xu Q, Xu N, Cheng Q, Qian Y, Huang R (2019). Epigenetically modulated FOXM1 suppresses dendritic cell maturation in pancreatic cancer and colon cancer. Mol Oncol.

[28] Condon TV, Sawyer RT, Fenton MJ, Riches DW (2011). Lung dendritic cells at the innate-adaptive immune interface. J Leukoc Biol.

[29] Dostert C, Grusdat M, Letellier E, Brenner D (2019). The TNF family of ligands and receptors: communication modules in the immune system and beyond. Physiol Rev.

[30] Di C, Lin X, Zhang Y, Zhong W, Yuan Y, Zhou T, Liu J, Xia Z (2015). Basophil-associated OX40 ligand participates in the initiation of Th2 responses during airway inflammation. J Biol Chem.

[31] Collison A, Hatchwell L, Verrills N, Wark PA, de Siqueira AP, Tooze M, Carpenter H, Don AS, Morris JC, Zimmermann N (2013). The E3 ubiquitin ligase midline 1 promotes allergen and rhinovirus-induced asthma by inhibiting protein phosphatase 2A activity. Nat Med.

[32] Doherty TA, Soroosh P, Khorram N, Fukuyama S, Rosenthal P, Cho JY, Norris PS, Choi H, Scheu S, Pfeffer K (2011). The tumor necrosis factor family member LIGHT is a target for asthmatic airway remodeling. Nat Med.

[33] Croft M, Duan W, Choi H, Eun SY, Madireddi S, Mehta A (2012). TNF superfamily in inflammatory disease: translating basic insights. Trends Immunol.

[34] Nocentini G, Ronchetti S, Petrillo MG, Riccardi C (2012). Pharmacological modulation of GITRL/GITR system: therapeutic perspectives. Br J Pharmacol.

[35] Placke T, Kopp HG, Salih HR (2010). Glucocorticoid-induced TNFR-related (GITR) protein and its ligand in antitumor immunity: functional role and therapeutic modulation. Clin Dev Immunol.

[36] Nocentini G, Riccardi C (2009). GITR: a modulator of immune response and inflammation. Adv Exp Med Biol.

[37] Chang YH, Wang KC, Chu KL, Clouthier DL, Tran AT, Torres Perez MS, Zhou AC, Abdul-Sater AA, Watts TH (2017). Dichotomous expression of TNF superfamily ligands on antigen-presenting cells controls post-priming anti-viral CD4(+) T cell immunity. Immunity.

[38] Chu KL, Batista NV, Wang KC, Zhou AC, Watts TH (2019). GITRL on inflammatory antigen presenting cells in the lung parenchyma provides signal 4 for T-cell accumulation and tissue-resident memory T-cell formation. Mucosal Immunol.

[39] Li W, Zhang L, Wu Z, Pickles RJ, Samulski RJ (2011). AAV-6 mediated efficient transduction of mouse lower airways. Virology.

[40] Kurosaki F, Uchibori R, Mato N, Sehara Y, Saga Y, Urabe M, Mizukami H, Sugiyama Y, Kume A (2017). Optimization of adeno-associated virus vector-mediated gene transfer to the respiratory tract. Gene Ther.

[41] Li L, Wen W, Jia R, Li Y, Liu X, Sun X, Li Z (2016). GITRL is associated with increased autoantibody production in patients with rheumatoid arthritis. Clin Rheumatol.

[42] Liu Y, Tang X, Tian J, Zhu C, Peng H, Rui K, Wang Y, Mao C, Ma J, Lu L (2014). Th17/Treg cells imbalance and GITRL profile in patients with Hashimoto's thyroiditis. Int J Mol Sci.

[43] Sukumar S, Wilson DC, Yu Y, Wong J, Naravula S, Ermakov G, Riener R, Bhagwat B, Necheva AS, Grein J (2017). Characterization of MK-4166, a clinical agonistic antibody that targets human GITR and inhibits the generation and suppressive effects of T regulatory cells. Cancer Res.

[44] Platania CBM, Ronchetti S, Riccardi C, Migliorati G, Marchetti MC, Di Paola L, Lazzara F, Drago F, Salomone S, Bucolo C (2020). Effects of protein-protein interface disruptors at the ligand of the glucocorticoid-induced tumor necrosis factor receptor-related gene (GITR). Biochem Pharmacol..

[45] Naura AS, Kim H, Ju J, Rodriguez PC, Jordan J, Catling AD, Rezk BM, Abd Elmageed ZY, Pyakurel K, Tarhuni AF (2013). Minocycline blocks asthma-associated inflammation in part by interfering with the T cell receptor-nuclear factor kappaB-GATA-3-IL-4 axis without a prominent effect on poly(ADP-ribose) polymerase. J Biol Chem.

[46] He D, Chen H, Zeng M, Xia C, Wang J, Shen L, Zhu D, Chen Y, Wang J (2020). Asthmatic airway vagal hypertonia involves chloride dyshomeostasis of preganglionic neurons in rats. Front Neurosci.

